# A Simple and Universal Nucleic Acid Assay Platform Based on Personal Glucose Meter Using SARS-CoV-2 N Gene as the Model

**DOI:** 10.3390/bios12040249

**Published:** 2022-04-15

**Authors:** Tian Li, Rui Pan, Yuhan Wen, Jiaqi Xu, Liping Zhang, Suna He, Gaofeng Liang

**Affiliations:** School of Basic Medical Sciences, Henan University of Science and Technology, Luoyang 471023, China; 191418030212@stu.haust.edu.cn (R.P.); 191418030221@stu.haust.edu.cn (Y.W.); 171418030124@stu.haust.edu.cn (J.X.); lipingzhang1826@haust.edu.cn (L.Z.); hesuna-2008@haust.cn (S.H.); lgfeng990448@haust.edu.cn (G.L.)

**Keywords:** nucleic acid assay, personal glucose meter, SARS-CoV-2, in vitro detection

## Abstract

A simple, selective, and quantitative platform for point-of-care diagnostic of COVID-19 is urgently needed as a complement in areas where resources are currently relatively scarce. To meet the needs of early diagnosis and intervention, a proof-of-concept demonstration of a universal personal glucose meter-based nucleic acid assay platform (PGM-NAAP) is presented, which converts to SARS-CoV-2 detection from glucose detection. By using magnetic bead separation together with the hand-held PGM for quantitative readout, PGM-NAAP achieves the 98 pM limit of detection for a sequence related to SARS-CoV-2. The ability to discriminate target nucleic acid from genomic DNA, the satisfactory spike recoveries of saliva and serum samples, as well as the good stability all together suggest the potential of the PGM-NAAP for the screening and diagnosis of suspected patients during the outbreaks of COVID-19 in resource-limited settings without sophisticated instruments. On the basis of these findings, PGM-NAAP can be expected to provide an accurate and convenient path for diagnosis of disease-associated nucleic acid.

## 1. Introduction

The COVID-19 has caused more than 426 million people being infected and no less than 5.9 million deaths as of February 2022. Recent studies have shown that at least 35% of patients are asymptomatic, which greatly increases the risk of epidemic transmission [1,2]. Since no specific medicine or treatment for COVID-19 is available, the accurate diagnosis of both symptomatic and asymptomatic cases, together with a series of prevention and control measures, including isolation and contact tracing, have become the most effective way to contain the outbreak [3,4,5].

Current diagnostic approaches for COVID-19 mainly include viral antigen detection and nucleic acid test [6,7,8,9]. Viral antigen detection can be realized directly in untreated biological fluids without virus lysis. Recently, several electrochemical platforms and lateral flow immunoassays (LFIA) were developed for detection of SARS-CoV-2 spike protein, which are suitable for massive diagnostic testing and home self-testing [10,11,12,13,14]. Unfortunately, since proteins cannot be amplified directly, detecting trace amounts of these proteins is still a considerable challenge and the selectivity maybe not good enough to distinguish similar viruses [1]. Due to the high sensitivity and specificity, the nucleic acid test is currently the most widely used diagnostic and screening tool for COVID-19, and RT-qPCR is the gold standard in clinical practice. However, it requires expensive equipment and well-trained personnel, which have made large-scale nucleic acid tests remain a challenge. To accelerate clinical diagnostic testing for SARS-CoV-2, it is quite necessary to develop point-of-care tests (POCT) because medical systems in many parts of the world have been overloaded during the pandemic [15,16,17].

As some of the existing techniques cannot be applied on a large scale in many scenarios, various diagnosis and screening strategies have been established for SARS-CoV-2 [18,19,20,21,22]. Most of these sensing strategies combine nucleic acid isothermal amplification with colorimetric or lateral flow-based detection strategies in order to reduce instrument dependence [23,24,25,26], achieving only qualitative or semiquantitative results as a compromise. The personal glucose meter (PGM) is recognized as one of the most successful POCT cases due to the reliable quantitative results, simple operation, small size, and wide acceptability all over the world. Traditionally, PGM is only applied to detect glucose in blood for the diagnosis and daily monitoring of hyperglycemia based on electrochemical detection or chemical colorimetry. For the first time, Lu’s group successfully used a PGM to detect non-glucose substances, which took full advantage of its simplicity and reliability [27,28,29,30]. Since then, this concept has been used to detect different kinds of targets, such as metal ion [31,32], toxins [33], viruses [34], bacteria [35], and disease biomarkers [36,37,38,39,40]. Recently, several groups have realized the detection of COVID-19 related biomarkers using the widespread, commercially available PGMs, with high sensitivity and specificity, which show good performance in clinical samples [6,20,41,42]. However, these experimental designs can hardly avoid the interference of endogenous glucose in patient samples. Recently, some electrochemical biosensors combining magnetic beads, sandwich hybridization assays, and enzymatic reaction have realized fast and sensitive nucleic acid detection [43,44].

Herein, in this work, we present a universal and quantitative PGM-based nucleic acid assay platform (PGM-NAAP). The nucleocapsid phosphoprotein gene (N gene) of SARS-CoV-2 was tested as the model, which can be recognized by the capture probes modified with invertase using sandwich-binding design. Then, the sandwich structure conjugates were separated and the invertase, which is directly related to the target concentration, could hydrolyze sucrose into glucose, thus the target could be determined by monitoring the glucose signal on the PGM. This reliable, simple, low-cost, and widely available PGM-NAAP makes it possible to carry out large-scale screening and diagnosis in communities and resource limited areas, effectively avoiding the overload of medical resources.

## 2. Materials and Methods

### 2.1. Reagents and Instruments

Sucrose, Na_2_HPO_4_, NaCl, and NaH_2_PO_4_ were sourced from Yongda Chemical Reagent Co., Ltd. (Tianjin, China). Tris (2-carboxyethyl)phosphine (TCEP) and diethylpyrocarbonate (DEPC)-treated water were from Sangon Biotech Co., Ltd. (Shanghai, China). Streptavidin-modified magnetic beads (10 mg/mL) were sourced from Thermo Fisher Scientific. Salmon sperm DNA and 4-(N-Maleimidomethyl)cyclohexane-1-carboxylic acid 3-sulfo-N-hydroxysuccinimide ester sodium salt (sulfo-SMCC) (>98%) were from Macklin Biochemical Co., Ltd. (Shanghai, China). Invertase (≥100 units/mg) was obtained from Yuanye Biochemical Co., Ltd. (Shanghai, China). Ultrafiltration tubes with molecular weight cut-off of 3, 30, and 50 kDa were purchased from Millipore Inc. Oligonucleotides used in this article were purchased from Sangon Biotech Co., Ltd. (Shanghai, China) and the corresponding sequences were as follows [45,46]:

Target: 5′-AGCAGTAGGGGAACTTCTCCTGCTAGAATGGCTGGCAATG-3′

Probe1: 5′-GGAGAAGTTCCCCTACTGCTA_20_/HS-3′

Probe2: 5′-biotin/A_10_CATTGCCAGCCATTCTAGCA-3′

Interference sequence 1: 5′-AGATGGGGGTTGAGGCTAAGCCGA-3′

Interference sequence 2: 5′-GCAGAAGCTGCAGGAGGTGAGCCGGATCTTGAAA CGGGTC-3′

SARS: 5′-GTCACGTATGTGCCATCCCAGGAGAGGAACTTCACCACAG-3′

MERS: 5′-CTCCTTAAACGGCAATGTTTCTACTGTTTTCGTGCCTGCA-3′

H1N1: 5′-GTAACAGTAACACACTCTGTTAACCTTCTAGAAG-3′

H7N9: 5′-ATTTTCAGCCGATTTAATTATTGAGAGGCGAGAA-3′

The PGM (ACCU-CHEK Active) was from Roche (Mannheim, Germany). The working principle of the PGM was provided in supporting information.

### 2.2. Preparation of Probe1−Invertase Conjugates

The Probe1−invertase conjugates were synthesized as described in the previous reports [29,38]. Thirty microliters of 100 μM sulfhydryl modified Probe1, 1 μL of 10 mM TCEP, and 2 μL of 1 M sodium phosphate buffer (pH = 5.3) was mixed and reacted at room temperature for 1 h. Then, the activated Probe1 was purified by ultrafiltration centrifuge tube (3 kDa) for eight times using buffer A (0.1 M sodium phosphate buffer, pH = 7.3). Meanwhile, 50 μL of 20 mg/mL invertase was activated with 1 mg sulfo-SMCC in buffer A at room temperature. One hour later, the insoluble sulfo-SMCC was first removed by centrifugation and the supernatant was further purified by an ultrafiltration centrifuge tube (50 kDa) for eight times using buffer A. Subsequently, the activated Probe1 and invertase were mixed and reacted at room temperature for 48 h. The product was then purified by an ultrafiltration centrifuge tube (50 kDa) eight times. Finally, the synthesized Probe1−invertase conjugates compound was diluted to 100 μL with buffer A and stored at 4 °C.

### 2.3. Procedures of PGM-NAAP

The detection of target nucleic acid was first carried out in buffer A. The assay mixture consisted of 1 μL of Probe1−invertase (10 mg/mL, the concentration of invertase represent the concentration of Probe1-invertase), 100 μL of target nucleic acid with concentration as indicated, and 1 μL of Probe2. Final concentrations of Probe1−invertase, Probe2, and target DNAs were 0.1 mg/mL, 100 nM, and 100 pM–20 nM, respectively. The mixture was cooled from 37 to 25 °C in 20 min to allow the DNAs to hybridize and form the sandwich structure (Probe1−invertase and target and Probe2). Then, 8 μL of streptavidin-conjugated magnetic beads (streptavidin-MBs) were added, and the mixture was kept at 25 °C for 30 min to allow the streptavidin-MBs to capture the sandwich structure. The assemblies, along with unattached MBs, were magnetically separated and washed three times with buffer A. Subsequently, 100 μL 1 M sucrose was added, and the mixture was kept at 37 °C for 120 min. The glucose signal was finally determined using the PGM and the sample-to-result turnaround time was roughly 180 min.

### 2.4. Detection of Target Nucleic Acid in Genomic DNA Using PGM-NAAP

The test was carried out in a large amount of salmon sperm DNA. The concentration of target nucleic acid was 10 nM and the mass ratio of salmon sperm DNA to target increased from 0 to 10^4^. The detection procedures were identical as mentioned above.

### 2.5. Detection of Target Nucleic Acid in Serum and Saliva Samples Using PGM-NAAP

In order to explore the feasibility of PGM-NAAP in clinical application, we tested it in fetal bovine serum and saliva. Healthy saliva samples (~500 μL) were collected in sterile tubes from volunteers. It is suggested to avoid eating 1 h before saliva collection, and rinse the mouth with water before sample collection [13].

Fetal bovine serum and saliva, diluted 10 times with buffer A, was filtered through an ultrafiltration centrifuge tube (30 kDa) and the filtrate were spiked with different concentrations of target nucleic acid separately. The detection process was the same as mentioned above.

### 2.6. Statistical Data Analysis

A statistical data analysis was performed using Statistical Program for Social Sciences (SPSS) ver. 22.0 software packages. Comparisons between the dependent variables were determined by using analysis of variance (ANOVA), and Fisher’s least significant difference (LSD). Results are expressed as mean value ± standard deviation (SD) of three determinations.

## 3. Results and Discussion

### 3.1. Principle of PGM-NAAP

**Scheme 1:**
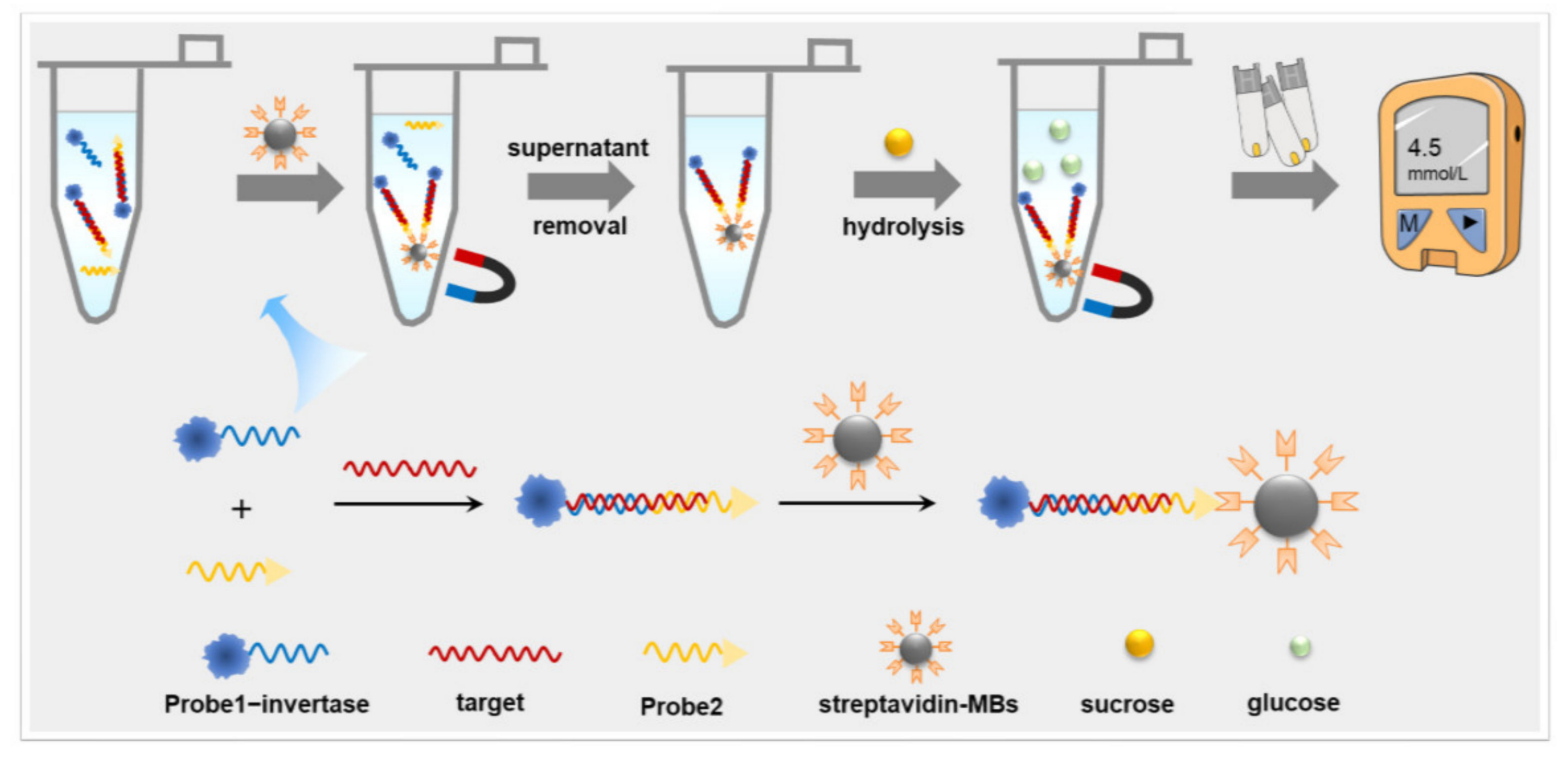
Illustration of the developed PGM-NAAP method.

The principle of PGM-NAAP is illustrated in Scheme 1. With the existence of target nucleic acid, a complex of “Probe1−invertase & target & Probe2” was formed through sandwich type DNA hybridization. The amount of invertase captured by magnetic beads was directly related to the target concentration. After magnetic separation and washing away of nonspecific adsorption, the bound DNA–invertase could catalyze the hydrolysis of sucrose, and the product, glucose, could be quantified easily by any untrained users with the help of a hand-held PGM. When compared with the existing SARS-CoV-2 detection methods based on PGMs [6,20,42], PGM-NAAP could effectively avoid the interference of endogenous glucose in the sample, since the sandwich structure conjugates with invertase were separated from the detection system and hydrolyzed sucrose after the washing step. That is to say, the invertase on the sandwich structure conjugates, which was directly related to the target concentration, hydrolyzed sucrose into glucose in a completely new and independent system, which has nothing to do with the original sample and the interferents in it. In general, the whole detection procedures of PGM-NAAP did not need any professional instruments or well-trained operators. As a consequence, the PGM-NAAP was expected to provide a simple and useful tool for POCT of COVID-19.

### 3.2. Design and Selection of Sequences Targeted for the N-Gene of SARS-CoV-2

During the current spread of SARS-CoV-2, research shows that the RdRP gene, E gene, and N gene of SARS-CoV-2 have conserved sequences. The N gene, which has 1260 nucleotide bases, was tested for the specific detection of SARS-CoV-2. To obtain better sensing performance, five different regions of N gene (40 mers) were selected as recognition sites and their capture probes were rationally designed separately. The capture probes and the target sequence could form a sandwich structure and the binding energy was predicted by NUPACK software for high detection efficiency (Table S1). Finally, the fragment (601–640) which had the lowest binding energy with its capture probes was chosen as a target model.

### 3.3. Optimization of Experimental Conditions

In order to obtain an optimal sensing performance, the reaction time, the amount of Probe1−invertase conjugation, the dosage of streptavidin-MBs, and incubation time were optimized.

The reaction time for invertase to hydrolyze sucrose has a very important impact on the performance of PGM-NAAP. The glucose concentration in the test tube was quantified by a PGM at intervals. For 20 nM target nucleic acid, the best signal-to-background ratio (SBR) was achieved with a reaction time of 120 min (Figure S1). Thus, 120 min was chosen in the following research.

The amount of Probe1-invertase conjugates is a crucial factor affecting the analytical performance of PGM-NAAP. On the one hand, enough Probe1-invertase conjugates are needed to capture the target nucleic acid. On the other hand, background signal may increase at high invertase levels due to nonspecific binding between the conjugates and magnetic beads, which in turn affects the sensitivity. For 10 nM target nucleic acid, the best SBR was obtained with 0.1 mg/mL Probe1−invertase conjugation (Figure S2). Hence, the optimal amount of Probe1−invertase conjugates was 0.1 mg/mL.

The dosage of streptavidin-MBs should be sufficient to capture and separate the sandwich structure (Probe1−invertase and target and Probe2). Five different dosages of streptavidin-MBs (0.1, 0.3, 0.5, 0.8, and 1 mg/mL) were used. As shown in Figure S3, the largest SBR was obtained at the dosage of 0.8 mg/mL and decreased with further increases in the amount of streptavidin-MBs. Thus, the dosage of streptavidin-MBs in the following study was 0.8 mg/mL.

The incubation time between the sandwich structure and streptavidin-MBs also influences the sensitivity of the PGM-NAAP. To study its effect, we detected 10 nM target nucleic acid with different incubation times, and found that the SBR initially increased from 15 to 30 min and then decreased (Figure S4). Therefore, incubation time was selected as 30 min in the following study.

### 3.4. Analytical Characteristics of the PGM-NAAP

**Figure 1:**
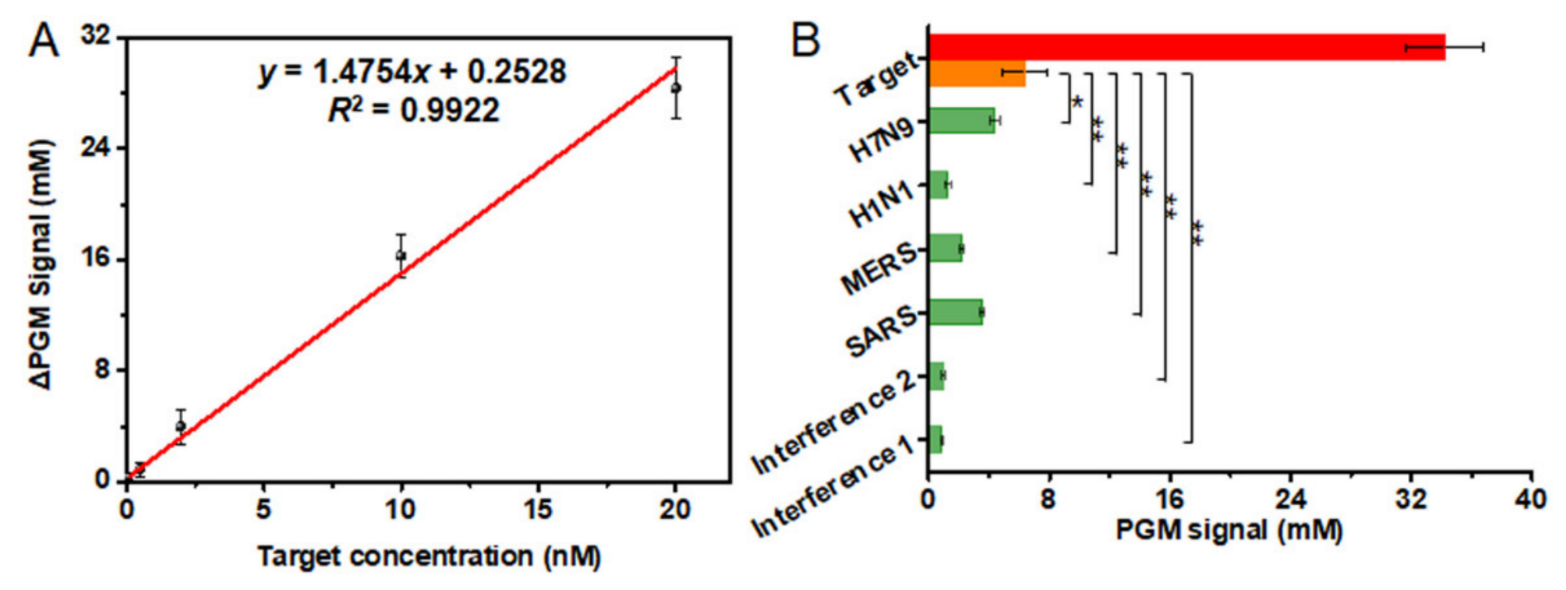
Detection performance of the PGM-NAAP: (**A**) the linear relationship between ΔPGM and the target concentration. The ΔPGM signal was obtained with the existence of different amounts of target sequence: 0.1 nM (6 × 10^7^ copy/μL), 0.5 nM (3 × 10^8^ copy/μL), 2 nM (1.2 × 10^9^ copy/μL), 10 nM (6 × 10^9^ copy/μL), and 20 nM (1.2 × 10^10^ copy/μL); (**B**) selectivity test. Interference 1 and interference 2 are random sequences with the length of 24 nucleotides and 40 nucleotides, respectively. The concentrations of target are 20 nM (1.2 × 10^10^ copy/μL, red bar) and 2 nM (1.2 × 10^9^ copy/μL, orange bar). The concentration of interference 1, interference 2, and the sequences related to SARS, MERS, H1N1, and H7N9 are 20 nM (1.2 × 10^10^ copy/μL). Tests were carried in three replicates (LSD; * *p* = 0.013, ** *p* < 0.001). Error bars represent the standard deviation (*n* = 3). For glucose detection, 1 mM equals 18 mg/dL.

The sensitivity of PGM-NAAP was first evaluated by measuring target nucleic acid at different concentrations under the optimized experimental conditions. The PGM signal change (ΔPGM) is defined as *P*_0_–*P*_1_, where *P*_0_ and *P*_1_ indicate the PGM signal in the absence and presence of target sequence, respectively. The background signal (*P*_0_) was (1.63 ± 0.048) mM (*n* = 10). As shown in Figure 1A, the ΔPGM increased with increasing concentration of target nucleic acid, and a good linear relationship (*R*^2^ = 0.9922) existed in the range from 0.1 to 20 nM, with a detection limit of 98 pM (5.9 × 10^7^ copy/μL, based on 3 *σ*/*k*, *n* = 10).

In the selectivity test, two random interference sequences and the sequences related to severe acute respiratory syndromes (SARS), Middle East respiratory syndrome (MERS), as well as the influenza viruses such as H1N1 and H7N9 were applied to the PGM-NAAP. A high PGM signal was obtained from the sample containing target N gene. In addition, as presented in Figure 1B, the observed PGM signal in the presence of target at 2 nM (1.2 × 10^9^ copy/μL) was statistically higher than those of the non-target sequence at 20 nM (*p* ≤ 0.013; LSD), confirming the high selectivity of PGM-NAAP.

**Figure 2:**
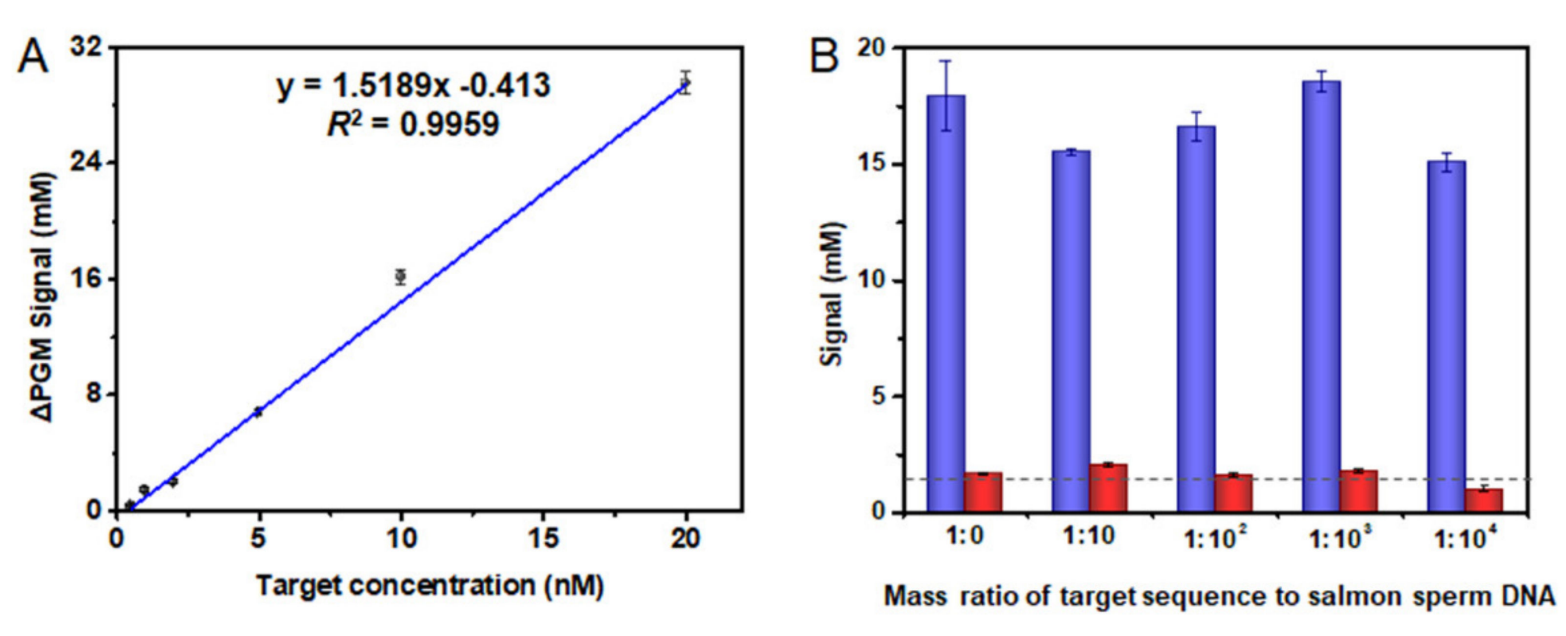
Target detection in salmon sperm DNA: (**A**) sensitivity test in 1 mg/mL salmon sperm DNA. The ΔPGM signal was obtained with the existence of different amounts of target sequence: 0.5 nM (3 × 10^8^ copy/μL), 1 nM (6 × 10^8^ copy/μL), 2 nM (1.2 × 10^9^ copy/μL), 5 nM (3 × 10^9^ copy/μL), 10 nM (6 × 10^9^ copy/μL), and 20 nM (1.2 × 10^10^ copy/μL).; (**B**) the signal value of the blood glucose meter changes with the quality of salmon sperm DNA in the system. The mass ratio of target sequence (N-gene) to salmon sperm DNA is 1:0 to 1:10^4^. The blue bar represents the concentration of N-gene at 10 nM (6 × 10^9^ copy/μL). The red bar is blank, and the dotted line is the average value of all blank signals. Error bars represent the standard deviation (*n* = 3). For glucose detection, 1 mM equals 18 mg/dL.

We further used salmon sperm DNA to simulate the complexity of the genome to verify the selectivity of PGM-NAAP. With the existence of 1 mg/mL salmon sperm DNA, a good linear relationship still existed between ΔPGM and the target nucleic acid concentration, with a detection limit of 0.2 nM (1.2 × 10^8^ copy/μL) (Figure 2A). Besides, the PGM signal did not fluctuate significantly as the mass ratio of genomic DNA to target increased from 0 to 10^4^. These results indicated that genomic DNA has little effect on target detection, which further confirmed the selectivity of PGM-NAAP.

### 3.5. Stability Test of the PGM-NAAP

**Figure 3:**
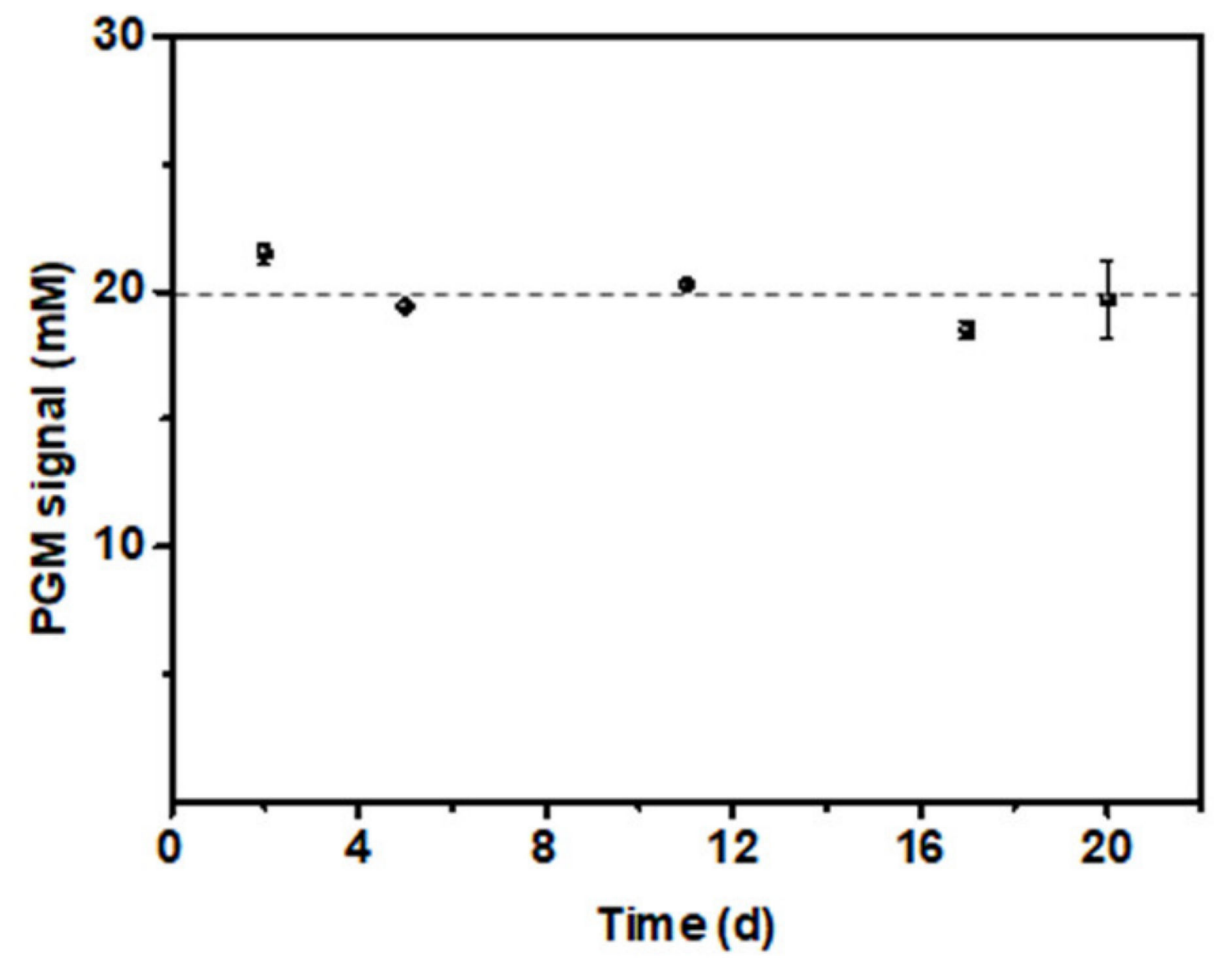
Stability test. The concentration of target sequence was 10 nM (6 × 10^9^ copy/μL). The dotted line is the average of 5 measurements. Error bars represent the standard deviation (*n* = 3). For glucose detection, 1 mM equals 18 mg/dL.

The stability of an assay is very important for clinical application and diagnosis. The prepared Probe1−invertase conjugates were kept at 4 °C, and five tests were conducted in the following 20 days for the detection of 10 nM (6 × 10^9^ copy/μL) target nucleic acid. No significant fluctuation was observed and the relative standard deviation of the five tests was calculated as 3.3%, indicating the good stability of both Probe1−invertase conjugates and PGM-NAAP (Figure 3).

### 3.6. Detection of SARS-CoV-2 Related Nucleic Acid in Biological Fluids

To explore the potential of PGM-NAAP in practical application, spike and recovery assays were carried out in fetal bovine serum, which is a complex matrix containing a variety of peptides, proteins, fats, carbohydrates, growth factors, hormones, inorganic substances, and so on. The diluted serum sample was spiked with two concentration levels of target and the spike recovery was (109.4 ± 12.4)% for the 100 pM target and (107.1 ± 6.7)% for the 20 nM target (*n* = 3).

**Figure 4:**
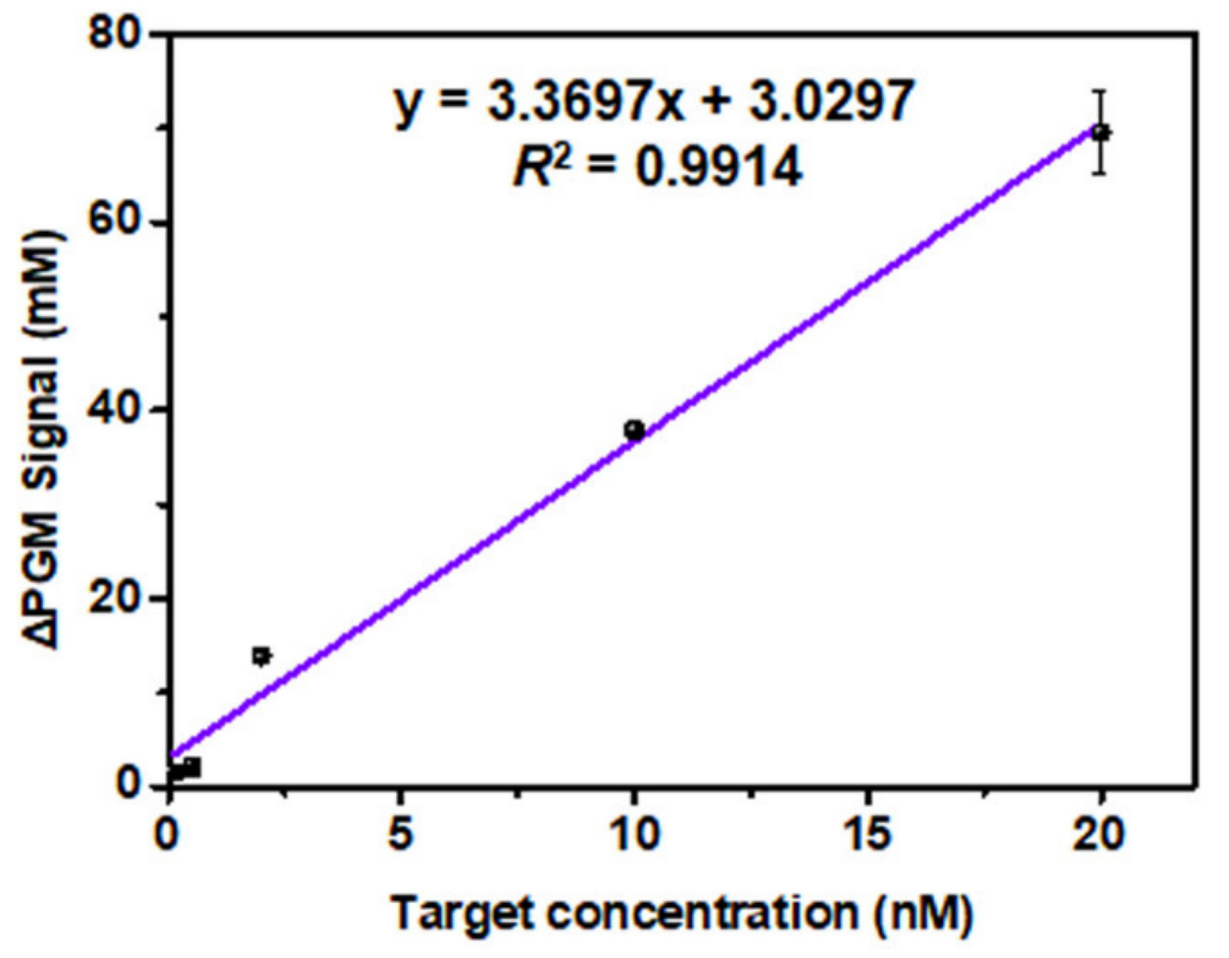
Application of the PGM-NAAP in 10% saliva. The ΔPGM signal was obtained with the existence of different amounts of target sequence: 0.1 nM (6 × 10^7^ copy/μL), 0.5 nM (3 × 10^8^ copy/μL), 2 nM (1.2 × 10^9^ copy/μL), 10 nM (6 × 10^9^ copy/μL), and 20 nM (1.2 × 10^10^ copy/μL). Error bars represent the standard deviation (*n* = 3). For glucose detection, 1 mM equals 18 mg/dL.

Previous research has shown that SARS-CoV-2 virus load is high in saliva [6], which is one of the most readily available and ideal human specimens for daily COVID-19 screening. So, we finally challenged the PGM-NAAP with the healthy saliva specimens spiked with different concentrations of target nucleic acid to prove the feasibility of the assay in a more real scenario. A calibration plot was generated with increasing target concentration from 0.1 to 20 nM (Figure 4). The LOD was calculated (3σ/slope) to be 89 pM (5.4 × 10^7^ copy/μL) in such a complex and viscous saliva sample. These preliminary results show that the PGM-NAAP is feasible for application in biological fluids.

## 4. Conclusions

In summary, we have successfully developed a simple and universal PGM-based nucleic acid assay platform with a detection limit of 98 pM for a sequence related to SARS-CoV-2. Since no sophisticated equipment is required, it provides the possibility for screening and early diagnosis of diseases in resource-limited areas such as healthcare centers and community hospitals. The ability to discriminate target nucleic acid from genomic DNA, and the satisfactory spike recoveries in saliva and serum samples, together with the good stability, all suggest the potential of the PGM-NAAP for monitoring suspected patients during COVID-19 outbreaks.

However, some limitations still exist for fully translating the current PGM-NAAP to a widely available TOC platform. The LOD is far from that obtained with PCR and there is no obvious advantage from the perspective of detection speed. Since the sandwich-binding design is versatile and can be easily expanded, pre-amplification should be adopted to achieve a higher sensitivity. A smartphone app, which can directly convert the PGM readout into the concentration of nucleic acid, should be developed to enhance the data progressing capability. In addition, the proposed PGM-NAAP contained several steps, which would be better to integrate into a one-pot reaction in potential by automation, minimal instrumentation, or a simplified process [47,48]. The above-mentioned limitations could be further investigated and solved by the future work. In conclusion, considering the universal experimental design and simplicity of PGMs, the PGM-NAAP has great potential to be used as a simple, quantitative, and portable sensing platform for the detection of other disease-related nucleic acids.

## Data Availability

The data presented in this study are available in Supplementary Materials.

## Supplementary Materials

The following supporting information can be downloaded at: https://www.mdpi.com/article/10.3390/bios12040249/s1, Table S1: Binding energy of different recognition site with their capture probes; Figure S1: Optimization of reaction time for invertase catalysis; Figure S2: Optimization of the amount of DNA-invertase conjugates; Figure S3: Optimization of the amount of magnetic beads; Figure S4: Optimization of the incubation time.
