# Supplementary material for: A Simple and Universal Nucleic Acid Assay Platform Based on Personal Glucose Meter Using SARS-CoV-2 N Gene as the Model
Source: Biosensors (Basel). 2022 Apr 15;12(4):249. doi: 10.3390/bios12040249 (PMC9025369; doi:10.3390/bios12040249)
Supplement: Supplementary file 1 [file biosensors-12-00249-s001.zip › biosensors-1654502-supplementary.pdf]

Supporting Information

A Simple and Universal Nucleic Acid Assay Platform Based on Personal Glucose Meter Using SARS-CoV-2 N Gene as the Model

Tian Li*, Rui Pan, Yuhan Wen, Jiaqi Xu, Liping Zhang, Suna He, and Gaofeng Liang

School of Basic Medical Sciences, Henan University of Science and Technology, Luoyang 471023, China.

Table of Contents

N gene sequence··S-2

Supplementary experimental information ···S-3

Supplementary Figures and Table··S-5

Working principle of the personal glucose meter···S-7

*Corresponding author:

E-mail address: litian90507@163.com

N-gene sequence

Target gene sequence of severe acute respiratory syndrome coronavirus 2 isolate Wuhan-Hu-1 gene="N" (1260 number of nucleotide bases)

Accession NC_045512

CDS 1..1260

/product="nucleocapsid phosphoprotein"

/protein_id="YP_009724397.2"

/translation="MSDNGPQNQRNAPRITFGGPSDSTGSNQNGERSGARSKQRRPQG
LPNNTASWFTALTQHGKEDLKFPRGQGVPINTNSSPDDQIGYYRRATRRIRGGDGKMK
DLSPRWYFYYLGTGPEAGLPYGANKDGIIWVATEGALNTPKDHIGTRNPANNAAIVLQ
LPQGTTLPKGFYAEGSRGGSQASSRSSSRSRNSSRNSTPGSSRGTSPARMAGNGGDAA
LALLLLDRLNQLESKMSGKGQQQQGQTVTKKSAAEASKKPRQKRTATKAYNVTQAFGR
RGPEQTQGNFGDQELIRQGTDYKHWPQIAQFAPSASAFFGMSRIGMEVTPSGTWLTYT
GAIKLDDKDPNFKDQVILLNKHIDAYKTFPPTEPKKDKKKKADETQALPQRQKKQQTV
TLLPAADLDDFSKQLQQSMSSADSTQA"

ORIGIN

1 atgtctgata atggacccca aaatcagcga aatgcacccc gcattacgtt tggtggaccc
61 tcagattcaa ctggcagtaa ccagaatgga gaacgcagtg gggcgcgatc aaaacaacgt
121 cggccccaag gtttacccaa taatactgcg tcttggttca ccgctctcac tcaacatggc
181 aaggaagacc ttaaattccc tcgaggacaa ggcgttccaa ttaacaccaa tagcagtcca
241 gatgaccaaa ttggctacta ccgaagagct accagacgaa ttcgtggtgg tgacggtaaa
301 atgaaagatc tcagtccaag atggtatttc tactacctag gaactgggcc agaagctgga
361 cttccctatg gtgctaacaa agacggcatc atatgggttg caactgaggg agccttgaat
421 acaccaaaag atcacattgg cacccgcaat cctgctaaca atgctgcaat cgtgctacaa
481 cttcctcaag gaacaacatt gccaaaaggc ttctacgcag aagggagcag aggcggcagt
541 caagcctctt ctcgttcctc atcacgtagt cgcaacagtt caagaaattc aactccaggc
601 agcagtaggg gaacttctcc tgctagaatg gctggcaatg gcggtgatgc tgctcttgct
661 ttgctgctgc ttgacagatt gaaccagctt gagagcaaaa tgtctggtaa aggccaacaa
721 caacaaggcc aaactgtcac taagaaatct gctgctgagg cttctaagaa gcctcggcaa
781 aaacgtactg ccactaaagc atacaatgta acacaagctt tcggcagacg tggtccagaa
841 caaacccaag gaaattttgg ggaccaggaa ctaatcagac aaggaactga ttacaaacat
901 tggccgcaaa ttgcacaatt tgcccccagc gcttcagcgt tcttcggaat gtcgcgcatt
961 ggcatggaag tcacaccttc gggaacgtgg ttgacctaca caggtgccat caaattggat
1021 gacaaagatc caaatttcaa agatcaagtc attttgctga ataagcatat tgacgcatac
1081 aaaacattcc caccaacaga gcctaaaaag gacaaaaaga agaaggctga tgaaactcaa
1141 gccttaccgc agagacagaa gaaacagcaa actgtgactc ttcttcctgc tgcagatttg
1201 gatgatttct ccaaacaatt gcaacaatcc atgagcagtg ctgactcaac tcaggcctaa

Optimization of reaction time for invertase catalysis

The experimental group consisted of 2 μL of Probe1−invertase (10 mg/mL, the concentration of invertase represent the concentration of Probe1−invertase), 100 μL of 20 nM target nucleic acid and 1 μL of 10 μM Probe2. The final concentrations of Probe1−invertase, Probe2, and target DNAs were 0.2 mg/mL, 100 nM, and 20 nM, respectively. The control group consisted of 2 μL of Probe1−invertase, 100 μL buffer A and 1 μL of 10 μM Probe2. The final concentrations of Probe1−invertase, Probe2, and target DNAs were 0.2 mg/mL, 100 nM, and 0, respectively. The mixture was cooled from 37 °C to 25 °C to allow the DNAs to hybridize and form the sandwich structure (Probe1−invertase & target & Probe2). Then 10 μL of 10 mg/mL streptavdin-conjugated magnetic beads (streptavidin-MBs) were added to the mixture and the mixture was kept at room temperature for 30 min to allow formation assemblies of streptavidin-MBs with the sandwich structure. The assemblies, along with unattached MBs was magnetically separated and washed with buffer A three times. Finally, 100 μL 1 M sucrose was added, and the mixture was allowed to react at 37 ℃ for 20 min, 40 min, 60 min, 80 min, 100 min,120 min and 140 min, respectively. The glucose content in each tube were finally measured using the PGM and the signal ratio of experimental group and control group was calculated respectively.

Optimization of the amount of DNA-invertase conjugates

In experimental group, 0.2 μL, 0.4 μL, 0.6 μL, 0.8 μL, 1 μL, 2 μL, 5 μL and 8 μL of Probe1−invertase (10 mg/mL, the concentration of invertase represent the concentration of Probe1−invertase) are diluted to 100 μL in different tubes. Then, 1 μL of 1 μM target nucleic acid and 1 μL of 10 μM Probe2 are added to each tube. In control group, 1 μL of 1 μM target nucleic acid was replaced of 1 μL buffer A. The dosage of Probe1−invertase and Probe2 are the same as those of the experimental group. The mixture was cooled from 37 °C to 25 °C to allow the DNAs to hybridize and form the sandwich structure (Probe1−invertase & target & Probe2). Then 10 μL of 10 mg/mLstreptavidin-MBs were added to the mixture and the mixture was kept at room temperature for 30 min to allow formation assemblies of streptavidin-MBs with the sandwich structure. The assemblies, along with unattached MBs was magnetically separated and washed with buffer A three times. Finally, 100 μL 1 M sucrose was added, and the mixture was allowed to react at 37 ℃ for 120 min. The glucose content in each tube were finally measured using the PGM and the signal ratio of experimental group and control group was calculated respectively.

Optimization of the amount of magnetic beads

The experimental group consisted of 1 μL of Probe1−invertase (10 mg/mL, the concentration of invertase represent the concentration of Probe1−invertase), 100 μL of 10 nM target nucleic acid and 1 μL of 10 μM Probe2. The final concentrations of Probe1−invertase, Probe2, and target DNAs were 0.1 mg/mL, 100 nM, and 10 nM, respectively. The control group consisted of 1 μL of Probe1−invertase, 100 μL buffer A and 1 μL of 10 μM Probe2. The final concentrations of Probe1−invertase, Probe2, and target DNAs were 0.1 mg/mL, 100 nM, and 0, respectively. The mixture was cooled from 37 °C to 25 °C to allow the DNAs to hybridize and form the sandwich structure (Probe1−invertase & target & Probe2). Then 1 μL, 3 μL, 5 μL, 8 μL and 10 μL of 10 mg/mL streptavidin-MBs were added to each tube and the mixture was kept at room temperature for 30 min to allow formation assemblies of streptavidin-MBs with the sandwich structure. The assemblies, along with unattached MBs was magnetically separated and washed with buffer A three times. Finally, 100 μL 1 M sucrose was added, and the mixture was allowed to react at 37 ℃ for 120 min. The glucose content in each tube were finally measured using the PGM and the

signal ratio of experimental group and control group was calculated respectively.

Optimization of the incubation time

The experimental group consisted of 1 μL of Probe1−invertase (10 mg/mL, the concentration of invertase represent the concentration of Probe1−invertase), 100 μL of 10 nM target nucleic acid and 1 μL of 10 μM Probe2. The final concentrations of Probe1−invertase, Probe2, and target DNAs were 0.1 mg/mL, 100 nM, and 10 nM, respectively. The control group consisted of 1 μL of Probe1−invertase, 100 μL buffer A and 1 μL of 10 μM Probe2. The final concentrations of Probe1−invertase, Probe2, and target DNAs were 0.1 mg/mL, 100 nM, and 0, respectively. The mixture was cooled from 37 ℃ to 25 ℃ to allow the DNAs to hybridize and form the sandwich structure (Probe1−invertase & target & Probe2). Then 8 μL of 10 mg/mL streptavidin-MBs were added to each tube and the mixture was kept at room temperature for 15 min, 30 min, 45 min, 60 min and 90 min, respectively. Then, the assemblies, along with unattached MBs was magnetically separated and washed with buffer A three times. Finally, 100 μL 1 M sucrose was added, and the mixture was allowed to react at 37 ℃ for 120 min. The glucose content in each tube were finally measured using the PGM and the signal ratio of experimental group and control group was calculated respectively.

Table S1 Binding energy of different recognition site with their capture probes

starting target position	ending target position	Probe1 (5'- 3')	Probe2 (5'- 3')	GC content	binding energy (kcal/mol)
421	460	CCAATGTGATCTTT TGGTGTA$_{20}$/HS	biotin/A$_{10}$TGTTAGCAG GATTGCGGGTG	47.5 %	-64.90
481	520	AATGTTGTTCCTTG AGGAAGA$_{20}$/HS	biotin/A$_{10}$CTGCGTAGA AGCCTTTTGGC	47.5 %	-65.48
601	640	GGAGAAGTTCCCC TACTGCTA$_{20}$/HS	biotin/A$_{10}$CATTGCCAG CCATTCTAGCA	52.5 %	-66.43
661	700	AATCTGTCAAGCA GCAGCAAA$_{20}$/HS	biotin/A$_{10}$TTTTGCTCTC AAGCTGGTTC	45.0%	-64.59
681	720	TTTTGCTCTCAAGC TGGTTCA$_{20}$/HS	biotin/A$_{10}$TTGTTGGCCT TTACCAGACA	45.0%	-64.89

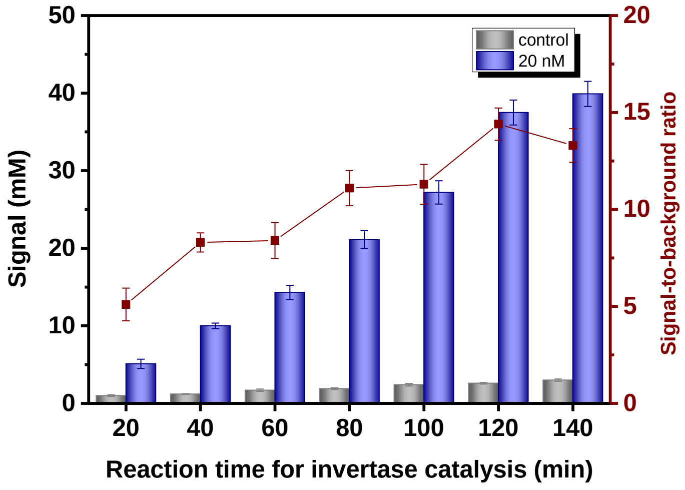

Figure S1. Optimization of reaction time for invertase catalysis

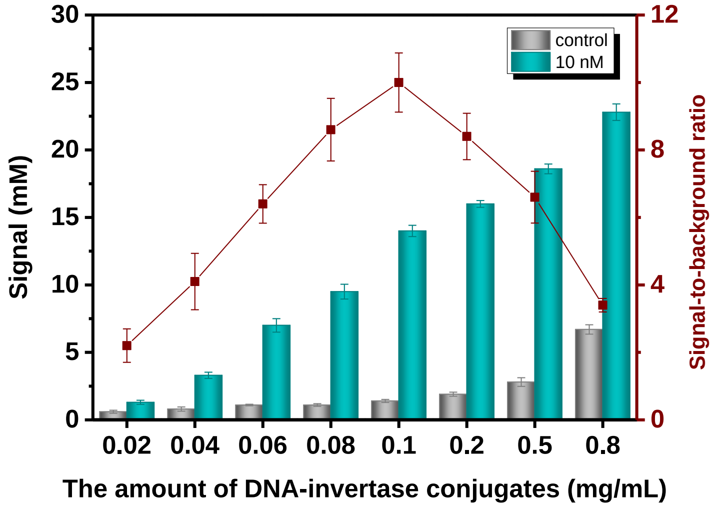

Figure S2. Optimization of the amount of DNA-invertase conjugates

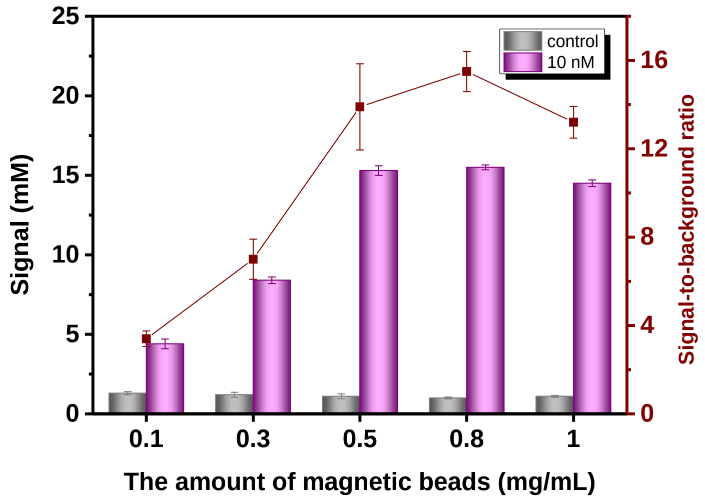

Figure S3. Optimization of the amount of magnetic beads

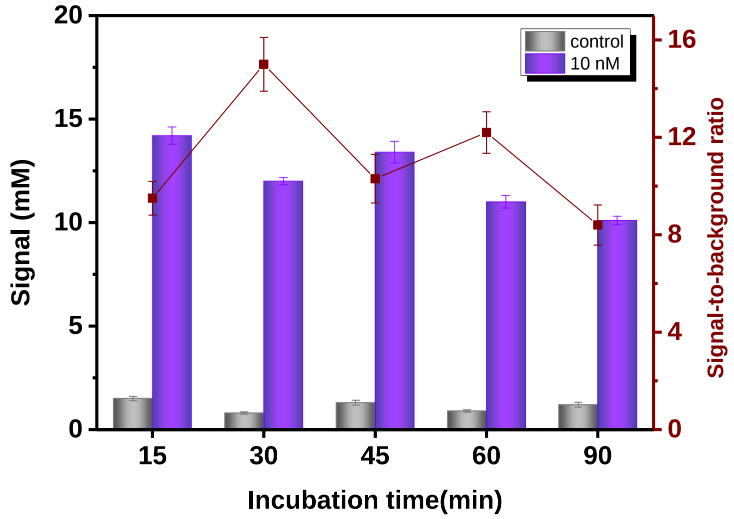

Figure S4. Optimization of the incubation time

Working principle of the personal glucose meter

The principle of the glucose meter (ACCU-CHEK® Active, Roche Diagnostics GmbH) used in this work is chemical colorimetry. Each test paper has a test area containing chemical reagents. When the sample is added to the test area, glucose dehydrogenase reacts with glucose. In the subsequent chemical reaction, the color of the test area changes. Based on the principle of photometer, the blood glucose meter can convert the obtained signal into glucose value according to the color change. The reaction process is as follows.

$$\beta\text{-D-Glucose} + \text{PQQ} \xrightarrow{\text{Mut.Q-GDH2}} \text{D-5-Gluconolactone} + \text{PQQH}_2$$

PQQ: pyrroloquinoline quinone Mut.Q-GDH2: mutant modified glucose dehydrogenase
